# Refractory Epilepsy in a Toddler With PPP2R1A Gene Mutation and Congenital Hydrocephalus

**DOI:** 10.7759/cureus.19988

**Published:** 2021-11-29

**Authors:** Samir Ruxmohan, Jonathan Quinonez, Randhir S Yadav, Shumneva Shrestha, Sujan Poudel, Joel D Stein

**Affiliations:** 1 Neurology, Larkin Community Hospital, Miami, USA; 2 Neurology/Osteopathic Neuromuscular Medicine, Larkin Community Hospital, Miami, USA; 3 Department of Internal Medicine, Institute of Medicine, Tribhuvan University, Kathmandu, NPL; 4 Department of Pediatrics, Tribhuvan University Institute of Medicine, Kathmandu, NPL; 5 Psychiatry and Behavioral Sciences, California Institute of Behavioral Neurosciences & Psychology, Fairfield, USA; 6 Division of Research & Academic Affairs, Larkin Community Hospital, South Miami, USA; 7 Osteopathic Neuromuscular Medicine, Family Medicine, Sports Medicine, Pain Medicine, Lake Erie College of Osteopathic Medicine (LECOM) Bradenton, Bradenton, FL, USA; 8 Pain Management, Osteopathic Neuromuscular Medicine, Sports Medicine, Larkin Community Hospital, South Miami, FL, USA

**Keywords:** refractory epilepsy, external ventricular drain, all neurology, developmental delay, ppp2r1a

## Abstract

Protein phosphatase 2A (PP2A) is a serine-threonine phosphatase that controls a variety of cellular functions. The *PPP2R1A* gene is present on chromosome 19 (19q13.41). Its mutation can interrupt B56δ-dependent dephosphorylation where B56δ is greatly expressed in the neural tissues. We present a case of a 14-month-old boy with infantile spasms, developmental delay, obstructive sleep apnea, PPP2R1A gene mutation, congenital hydrocephalus, hypoplastic/absent corpus callosum, pontocerebellar hypoplasia, and medically refractory seizures. He underwent multiple surgical procedures that include endoscopic third ventriculostomy with choroid plexus cauterization, ventriculoperitoneal shunting, and external ventricular drain for progressive hydrocephalus with multiple antiepileptic regimes for refractory epilepsy with variable response.

## Introduction

Epilepsy is a common neurological disorder of major health concern. Globally, 1% of the population has active epilepsy [1]. A population-based study estimated 10% of the population will have at least one episode of seizure during their lifetime [2]. Even in countries with adequate facilities to diagnose and treat, 30-40% of people with epilepsy are noted to have uncontrolled seizures [1].

## Case presentation

We present a case of a 14-month-old male with a past medical history of congenital hydrocephalus, infantile spasms, developmental delay, PPP2R1A gene mutation, obstructive sleep apnea, and medically refractory seizures who presented to a local hospital for evaluation of an increased seizure frequency, fever, and non-bilious/non-bloody emesis of four days duration. His past surgical history consisted of prior interventions that included a choroid plexus cauterization and a ventriculoperitoneal placement. On physical examination, he had macrocephaly with frontal bossing. He was awake with eyes open with upward gaze preference. There were intermittent non-purposeful movements of all extremities, decreased muscle bulk, axial hypotonia with distal extremity hypertonia, arching with slight opisthonic posturing and reflexes were 3+ bilaterally. An MRI of his brain without contrast showed hypoplastic/absent corpus callosum, pontocerebellar hypoplasia. A video electroencephalogram (vEEG) demonstrated showed epileptiform discharges from the left central region and recording consistent with epileptiform encephalopathy. In addition, a videofluoroscopic swallow study (VFSS) done showed functional airway protection with thin liquids. He was placed on levetiracetam and phenobarbital and admitted for further management.

After admission, given the past few days of increased seizure frequency, a prolonged seizure on admission, lacosamide was loaded and continued on maintenance dose with no recurrent seizure. He tolerated the new anti-epileptic regimen. With a concern for ongoing breakthrough seizures, he was placed back on vEEG for further characterization. Compared to the last one done about four months back, the vEEG showed worsening of epileptiform discharges; a bilateral independent region with severe epileptic encephalopathy. Consequently, a medication adjustment with clobazam was made. His parents requested to wean off levetiracetam as they didn’t believe it has helped, so clobazam was further optimized and levetiracetam was weaned off. He was also prescribed with rescue medicine diazepam per rectal and clonazepam as needed on sick days. He was also kept on cannabidiol and a ketogenic diet.

He was later taken to the operating room for removal of right strata ventroperitoneal shunt (VPS) due to meningitis/encephalitis [cerebrospinal fluid (CSF) culture showed methicillin-susceptible Staphylococcus aureus) and insertion of a tunneled external ventricular drain (EVD); however, an MRI of his brain without contrast gave concern for EVD dislodgement. He underwent another exploratory surgery to ensure that his EVD was in place and had a repeat MRI one week later which demonstrated an intact EVD.

As this patient already was diagnosed with a PP2R1A gene mutation prior to presentation, management during her hospital course focused solely on seizure prevention and EVD dislodgement. No genetic analysis was indicated for further evaluation of the patient's increased seizure frequency. 

## Discussion

PP2A is a serine-threonine phosphatase that regulates a variety of cellular functions. It is a heterotrimeric protein composed of (A) a scaffolding subunit, (B) variable regulatory subunits, and (C) a catalytic subunit [3]. The PPP2R1A gene is present on chromosome 19 (19q13.41) which encodes isoform α of the scaffolding subunit (Aα). The Aα scaffolding subunit functions to link the catalytic and regulatory subunits including B56δ [4]. Moreover, mutations in the PPP2R1a gene can interrupt B56δ-dependent dephosphorylation [4] where B56δ is greatly expressed in the neural tissues [4,5]. Seven cases of de novo mutations in the PPP2R1A gene have been described in the literature with predominant features of agenesis or hypoplasia of corpus callosum, hypotonia, developmental delay, severe intellectual disability, ventriculomegaly, and dysmorphic features [4-7]. These features were consistent with findings in our patient. Most recently, a broad phenotypic spectrum of PPP2R1A-related neurodevelopmental disorder in 30 individuals found language delay, hypotonia, and hypermobile joints along developmental delay ranging from mild learning to severe intellectual disabilities (ID) with or without epilepsy. Individuals without B55α subunit-binding deficit had macrocephaly, less severe ID, and no seizures while impaired B55α but increased striatin binding were found biochemically more disruptive variants with associated profound ID, epilepsy, hypoplasia of corpus callosum, and occasionally microcephaly [8].

Hydrocephalus is a condition of increased intraventricular pressure and pathological dilatation of ventricles which has significantly caused morbidity and mortality in children [9]. Among the myriad of clinical features, Cushing’s triad (hypertension, bradycardia, and irregular respiration) is the most common presentation but may vary according to the age of presentation [10]. These were consistent in our patient. Besides, some late manifestations like Parinaud syndrome (dorsal midbrain syndrome) and new-onset seizures necessitate urgent intervention [10]. A seizure should be considered a sign of an advanced form of hydrocephalus if there is no other explainable cause [10]. Thus, intervention for advanced stage hydrocephalus is required [10]. Imaging studies are useful in diagnosing hydrocephalus while serial head circumference measurement can help monitor the progress [10]. In children showing progressive features of hydrocephalus, CSF diversion procedures remain the standard of care where the ventricular shunt is the most common one [10]. Alternately, newer endoscopic minimally invasive procedures can also be used [10]. The five-year outcome in International Infant Hydrocephalus Study (IIHS) showed a better overall health status and quality of life in the endoscopic third ventriculostomy (ETV) cohort but no significant difference between those treated initially with ETV or shunt [11-14]. The immediate risks of surgical procedures for hydrocephalus include strokes, intraparenchymal or subdural hemorrhage, catheter misplacement causing nonfunctional shunt, or urgent reoperation [10,15-19]. In our patient, hydrocephalus was confirmed on MRI. Further, surgical procedure ETV/choroid plexus catheterization (CPC) was done to drain the CSF. He developed meningitis/encephalitis so EVD was placed later. But it also got dislodged which was repositioned.

## Conclusions

Managing seizures can be a great challenge in a child with PPP2R1A gene mutation and congenital hydrocephalus. Different standard medical and surgical treatments may show variable effects on symptom control and disease progression.
